# The First Thousand Days: Kidney Health and Beyond

**DOI:** 10.3390/healthcare9101332

**Published:** 2021-10-06

**Authors:** Chien-Ning Hsu, You-Lin Tain

**Affiliations:** 1 Department of Pharmacy, Kaohsiung Chang Gung Memorial Hospital, Kaohsiung 833, Taiwan; cnhsu@cgmh.org.tw; 2 School of Pharmacy, Kaohsiung Medical University, Kaohsiung 807, Taiwan; 3 Department of Pediatrics, Kaohsiung Chang Gung Memorial Hospital and Chang Gung University College of Medicine, Kaohsiung 833, Taiwan; 4 Institute for Translational Research in Biomedicine, Kaohsiung Chang Gung Memorial Hospital and Chang Gung University College of Medicine, Kaohsiung 833, Taiwan

**Keywords:** Developmental Origin of Health and Disease (DOHaD) theory, congenital anomalies of the kidney and urinary tract (CAKUT), kidney disease, nitric oxide, infant, pregnancy, children, oxidative stress, renin–angiotensin system

## Abstract

The global burden of chronic kidney disease (CKD) is rising. A superior strategy to advance global kidney health is required to prevent and treat CKD early. Kidney development can be impacted during the first 1000 days of life by numerous factors, including malnutrition, maternal illness, exposure to chemicals, substance abuse, medication use, infection, and exogenous stress. In the current review, we summarize environmental risk factors reported thus far in clinical and experimental studies relating to the programming of kidney disease, and systematize the knowledge on common mechanisms underlying renal programming. The aim of this review is to discuss the primary and secondary prevention actions for enhancing kidney health from pregnancy to age 2. The final task is to address the potential interventions to target renal programming through updating animal studies. Together, we can enhance the future of global kidney health in the first 1000 days of life.

## 1. Introduction

The first 1000 days of life, the period from conception to two years of age, is crucial for the individual’s later development. Our body can adapt in response to stimuli from the environment through alterations of structure or function, namely, developmental plasticity [1]. Suboptimal environmental conditions in this unique period can cause adverse long-term health outcomes. This theory has now evolved into the Developmental Origin of Health and Disease (DOHaD) theory [2]. These environmental factors include, but are not limited to, nutrition, maternal illness, environmental chemicals, substance abuse, medication use, infection, and exogenous stress [3]. Recent advances in epidemiological and experimental studies have offered considerable insight into how various environmental influences during early development increase the risk for developing chronic, especially non-communicable, disease (NCD), in later life [4].

Chronic kidney disease (CKD) is one of the most prevalent NCDs [5]. More importantly, CKD is a key determinant of poor health outcomes for major NCD [6]. The global burden of CKD is rising and now affects 10% of the world’s adult population [7]. CKD can originate in early life. A variety of adverse environmental conditions are associated with the programming of kidney disease [8,9,10]. We now know that programming processes before disease becomes apparent are modifiable by shifting the therapeutic approach from adulthood to early life, namely, reprogramming [11,12]. Accordingly, this vision proposes that greater attention is needed on global kidney health policy, particularly focus on the prevention of kidney disease in the earliest stage, not just the treatment of established CKD [13]. Thus, this review places specific emphasis on gaining a greater understanding of the pathophysiological phenomenon behind the programming of kidney disease and current evidence relating to preventing CKD in the first 1000 days of life by developing a potential reprogramming strategy. Our search strategy was designed to retrieve literature relating to DOHaD and kidney disease from PubMed/MEDLINE databases. Specific emphasis was placed on environmental insult exposure during pregnancy, lactation, and infant stages. Additional studies targeting the pathogenesis of developmental programming of kidney disease were also considered.

## 2. Biological Processes Shaping Kidney Development

**Figure 1:**
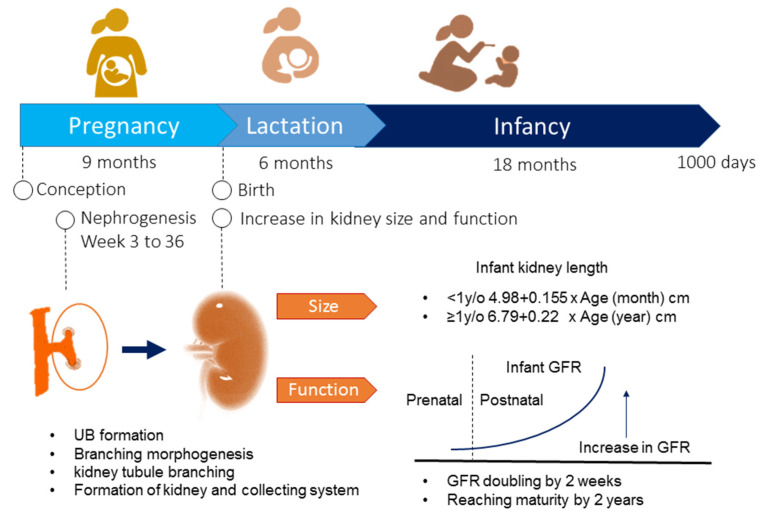
Kidney development during the first 1000 days of life. UB = ureteric bud; GFR = glomerular filtration rate.

Figure 1 illustrates the biological processes of kidney development during the first 1000 days of life. In humans, kidney development begins at week three and ends at around 36 weeks of gestation [14]. The metanephric kidney is initiated when the ureteric bud (UB) forms and elongates to invade the adjacent metanephric mesenchyme (MM) [15]. The MM forms nephrons, while the UB tip branches serially to form the collecting duct. The renal vesicles form by a mesenchyme to epithelium conversion and are the precursors of the nephrons. Branching morphogenesis establishes an extensive ureteric bud arborization [15], which eventually differentiates into the collecting duct system and leads to the formation of the nephrons. A nephron is the basic functional unit of the kidney. The human kidneys are composed of approximately 1 million nephrons, with a 10-fold interindividual variability [14]. An exponential increase in nephrons occurs between 18 and 32 weeks of gestation. By the end of gestation, nephron development is complete [16]. In general, nephrogenesis is complete by term birth.

After birth, the kidney continues to grow in size. For infants younger than 1 year, the equation is as follows: renal length (cm) = 4.98 + 0.155 X age (months) [17]. For babies older than 1 year, the regression equation is as follows: renal length (cm) = 6.79 + 0.22 X age (years) [17]. Regarding renal function, the glomerular filtration rate (GFR) doubles in the first 2 weeks of life from a value of 20 mL/min/1.73 m^2^ at birth in full-term neonates. The GFR continues to increase after birth and reaches adult values by two years of age [18].

## 3. Risk Factors Influencing Kidney Health and Development

Branching morphogenesis is critical for a normal nephron number [15]. Impaired branching morphogenesis and nephrogenesis could cause a reduced nephron number and a broad spectrum of malformed kidneys, namely, congenital anomalies of the kidney and urinary tract (CAKUT) [19]. Developing kidneys are vulnerable to environmental risk factors that impair development during pregnancy: a severe renal maldevelopment occurs during early pregnancy, while kidney defects that occur later are generally less severe [19]. A case-control study recruiting more than 1.6 million infants demonstrated that risk factors for CAKUT consist of prematurity, low birth weight (LBW), male sex, maternal gestational diabetes, maternal thalassemia, oligohydramnios or polyhydramnios, and first parity [20].

Human and experimental studies suggest genetic factors, including chromosomal anomalies, copy number variants, and monogenic mutations/deletions, likely contribute to approximately 45% of CAKUT. Environmental factors and not yet identified genetic factors contribute to the remainder [19,21,22]. Although hundreds of candidate genes have been identified, CAKUT cannot be attributed to a monogenic cause in more than 80% of cases [19]. Considering the phenotypes from genetic defects vary considerably, this suggests that many cases of CAKUT are polygenic. Notably, gene–gene and gene–environment interactions have also contributed to CAKUT [23]. One example is apolipoprotein L1 (APOL1) variants [24,25]. Prior research suggests that environmental stressors and APOL1 may contribute to the CKD phenotype variance associated with APOL1 risk alleles. Under basal conditions, genotypes carrying risk alleles appear to have a subtle phenotype that is not disease causing. However, high-risk genotypes cannot adapt to the stresses and lose renal function, resulting in CKD throughout the entire lifespan [25]. Additionally, genes are vulnerable to epigenetic modification in response to adverse conditions during the first 1000 days of life. Likewise, epigenetic changes may provide a mechanistic link whereby early life exposures lead to long-term increased risk of kidney disease in adulthood.

As nephrogenesis is completed by full-term birth, premature infants tend to develop low nephron endowment. Additionally, a low nephron number is related to compromised pregnancy, low birth weight, intrauterine growth retardation, inadequacy of postnatal nutrition, and treatment with nephrotoxic drugs after birth, etc. [9,16,26]. The role of low nephron number in renal programming is gaining attention as it can cause glomerular hyperfiltration and compensatory glomerular hypertrophy, and can initiate a vicious cycle of further reductions in nephrons [16]. As kidney disease is possibly the result of interactions among multiple hits [27], a low nephron number may create a first hit to the kidney, which increases the vulnerability of remaining nephrons to develop CKD when facing other environmental insults as a second hit in later life.

**Figure 2:**
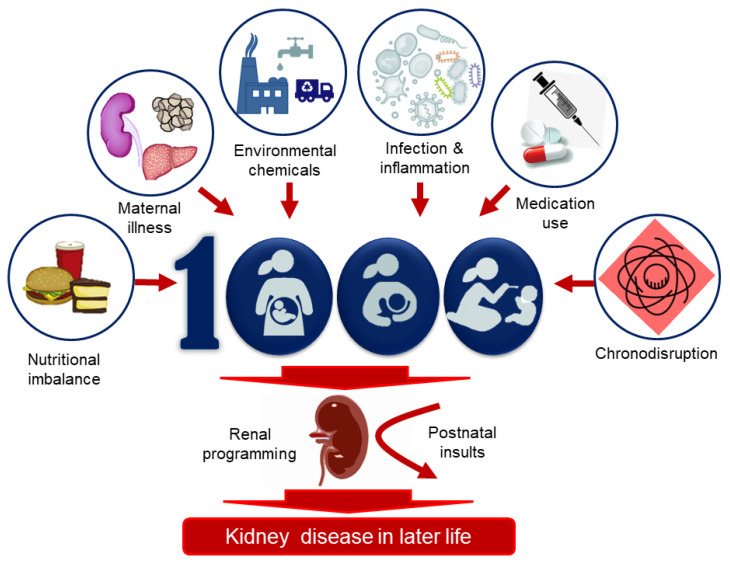
Various environmental factors during the first 1000 days of life are associated with renal programming, resulting in kidney disease in later life. These risk factors include nutritional imbalance, maternal illness, environmental chemicals, infection and inflammation, medication use, and chronodisruption.

Thus far, several environmental risk factors have been linked to the programming of kidney disease, including nutritional imbalance, maternal illness, environmental chemicals, substance abuse, medication use, infection, and exogenous stress (Figure 2). These are discussed in turn.

### 3.1. Nutritional Imbalance

The insufficient or excessive consumption of certain nutrients has been linked to the developmental programming of kidney disease [28,29,30]. Important support was first provided by the Dutch famine birth cohort study for renal programming, which demonstrated that undernutrition during pregnancy has long-lasting consequences for adult health, including kidney disease [31]. Several epidemiological studies have associated maternal nutrition during pregnancy with offspring kidney structure and function in humans, as reviewed elsewhere [29]. Deficiencies in maternal folate [32], vitamin A [33], and total energy [31] during pregnancy were associated with negative impacts on kidney structure and function, measured by kidney volume, proteinuria, and renal function in the offspring [29].

There is convincing evidence from animal studies of nutritional imbalance during pregnancy and lactation affecting renal programming, resulting in kidney disease in adult offspring. Various nutritional factors have been related to renal programming, including calorie restriction [34], protein restriction [35], low-salt intake [36], magnesium-deficient diet [37], high sucrose consumption [38], high-fructose diet [39], high-fat diet [40], and high-salt diet [41].

Additionally, nutritional imbalance during pregnancy and lactation is also associated with a reduced nephron number, a key determinant of adulthood kidney disease. In rat models, nutritional insults last only for a brief period, as little as 1–2 days, but can impair kidney development, resulting a permanent low nephron endowment [9]. Currently, a variety of animal models of early-life suboptimal nutrition, such as maternal caloric restriction [42], low protein diet [43], vitamin A deficiency [44], multi-deficient diet [45], iron restriction diet [46], low-salt diet [47], and high-salt diet [47], have been reported to impair nephrogenesis, resulting in a reduced nephron number.

### 3.2. Maternal Illness

Maternal illnesses and complications during pregnancy can drive renal programming and increase the risk for developing kidney disease later in life. Several animal models resembling human illnesses and pregnancy complications have been established to study renal programming-related offspring outcomes, such as hypertensive disorders of pregnancy [48], preeclampsia [49], CKD [50], diabetes [51], and sleep disorder [52]. Hypertension affects up to 10% of pregnancies [53]. In spontaneously hypertensive rats, maternal hypertension is related to renal programming and hypertension in the adult offspring [48]. Another study showed pregnant rats treated with N^G^-nitro-L-arginine-methyl ester (L-NAME, a nitric oxide synthase inhibitor) to mimic maternal preeclampsia caused elevated blood pressure (BP) and renal programming in their adult offspring [49]. To study the influence of maternal CKD on offspring’s renal outcome, we used an adenine-induced maternal CKD model. Our findings indicated that uremia-related adverse outcomes in adult offspring included renal hypertrophy and hypertension [54].

Diabetes in pregnancy is also thought to impair nephrogenesis. Human studies have demonstrated that adults born to mothers with gestational diabetes have an increased risk of CAKUT [20,55] and kidney disease [56]. Another observational study showed that diabetes during pregnancy influences fetal kidney growth, indicating a negative effect on nephrogenesis [56]. In a streptozotocin-induced diabetes rat model, offspring born to diabetic dams developed a reduced nephron number, renal hypertension, and kidney injury [51]. Moreover, sleep disorder in pregnancy also affects kidney development. In a maternal sleep restriction model, adult offspring displayed an enlarged glomeruli diameter and a reduced number of glomeruli coinciding with hypertension at 2 months of age [52]. These findings suggest that sleep restriction during pregnancy impairs nephrogenesis, resulting in renal programming-related disorders in offspring.

From human and animal studies, maternal obesity is another risk factor for CKD in offspring [57]. An observational study recruiting 3093 CAKUT cases showed a positive association between maternal obesity and CAKUT in offspring [58]. Another meta-analysis study supports the notion that maternal obesity adversely impacts renal programming in offspring, with an increased risk of kidney disease in adulthood [59]. In various animal models of maternal obesity, offspring from obese mothers had higher serum creatinine levels, a 24 h urinary albumin to creatinine ratio, and worse renal tubular injury and glomerulosclerosis scores [40,60,61]. In view of the alarming increase in global obesity, more attention should be paid to studying how maternal obesity influences CKD development in offspring.

### 3.3. Environmental Chemicals

Numerous environmental chemicals pose a wide range of adverse effects on the kidney [62]. During kidney development, some chemicals can impair nephrogenesis, leading to low nephron endowment and CAKUT [63]. Accordingly, developmental nephrotoxic effects can be expected during the environmental chemical exposure of pregnant women. After birth, infants can still be at an increased risk of nephrotoxicity to elemental (e.g., mercury) or organic contaminants (e.g., melamine) [64,65].

There are several observational studies addressing the implication of maternal environmental chemical exposure in offspring’s renal outcome. Two studies investigated the associations between maternal lead levels and renal outcomes in offspring [66,67]. One study found there were no associations between maternal lead levels and estimated GFR (eGFR) at 8–12 years of age. However, they observed that the maternal lead level was negatively associated with kidney volume in children [66]. Another study reported there was an inverse association between maternal blood lead levels and eGFR in overweight children at 8–12 years of age [67]. Epidemiological studies revealed that maternal exposure to polycyclic aromatic hydrocarbon, per- and polyfluoroalkyl substances, phthalates, polycyclic aromatic hydrocarbon, and PM_2.5_/PM_10_ associated with preterm birth and LBW [68,69,70,71,72], are both risk factors related to a low nephron number.

Evidence from experimental studies also support that exposure during pregnancy can affect kidney development, resulting in renal programming. Maternal exposure to 2,3,7,8-tetrachlorodibenzo-p-dioxin (TCDD) or bisphenol A causes a rise in BP in adult rat offspring [73,74], which is relevant to renal programming. Additionally, hydronephrosis was described in rat offspring prenatally exposed to TCDD [75]. Animal studies of the implication of maternal heavy metal exposure in the kidneys of offspring suggested that cadmium is the main cause of adverse renal outcomes [76].

### 3.4. Substance Abuse

As with nutrient and chemical effects during kidney development, substance abuse is also a major maternal insult. In the United States, roughly 6–16% of pregnant women are cigarette smokers, alcohol abusers, or illicit drug users [77]. An observational study has shown that maternal alcohol exposure has a dose-dependent adverse effect on renal function in overweight and obese children [78]. Another cohort study revealed that maternal alcohol exposure is associated with the development of mild CKD in their offspring at 30 years [79]. Similarly, in a maternal ethanol exposure rat model, reduced nephron number and renal function were reported in adult offspring, possibly as a result of inhibited ureteric branching morphogenesis [80].

In humans, maternal smoking during pregnancy is associated with fetal and infant kidney volume [81]. Likewise, prior research on animal models demonstrated that maternal nicotine exposure adversely affected fetal kidney development, resulting in CKD in offspring [82,83,84]. Though illicit drug use is associated with a higher risk of CKD progression [85], whether maternal illicit drug use affects offspring’s renal outcomes remains largely unknown.

### 3.5. Infection and Inflammation

Intrauterine infection is a crucial and frequent mechanism leading to preterm birth [86]. Microbial endotoxins and proinflammatory cytokines stimulate the production of prostaglandins, resulting in uterine contractility [87]. Furthermore, emerging evidence indicates that maternal infections contribute to poor birth outcomes, such as LBW and preterm birth, by the inflammation-mediated disruption of placental development and function [88].

The influence of infection during gestation on offspring’s renal outcomes has been studied in animal models. Maternal exposure to lipopolysaccharide (LPS) caused offspring hypertension coinciding with renal programming [89,90]. Another study revealed that prenatal LPS exposure augmented neonatal hyperoxia-induced kidney injury [91].

After birth, urinary tract infection (UTI) is one of the major infections contributing to adverse renal outcomes [92]. Pyelonephritis can lead to renal scarring and result in hypertension and even kidney failure. Approximately 30 % of children who develop a UTI are subsequently diagnosed with vesicoureteral reflux [93]. Reflux nephropathy is reported as the fourth most frequent cause of end-stage kidney disease in the pediatric population [92].

### 3.6. Medication Use

The existing literature suggests that a number of drugs administrated to pregnant women may affect kidney development, leading to CAKUT [84]. These medications include, but are not limited to, aminoglycosides, cyclosporine A, NSAIDs, ACE inhibitor (ACEI)/angiotensin receptor blockers (ARBs), dexamethasone, furosemide, anti-epileptic drugs, Adriamycin, and cyclophosphamide [94]. In various animal models, cyclosporine A [95], gentamicin [96], and glucocorticoid [97,98,99] have been associated with a low nephron number and renal programming [9].

Most nephrotoxic drugs in mature kidneys may also have toxic effects on developing kidneys. It is worth noting that drugs that are not nephrotoxic in fully developed kidneys may impair the balance of growth factors that are crucial for kidney development. For example, ACEI/ARBs are well known to exert renoprotective benefits [100]. However, these drugs have been avoided in pregnant women due to ACEI/ARB fetopathy and renal maldevelopment [101]. The reason for this is that the suppression of the intrarenal RAS contributes to altered structural development of the kidney [102]. Another example is glucocorticoid. Currently, antenatal glucocorticoid administration is recommended in women at risk of preterm birth to accelerate fetal lung maturation [103]. In normal pregnancy, the fetus is protected by the placental inactivation of active glucocorticoids [104]. Accordingly, excessive glucocorticoid through exogenous administration has been related to renal programming, resulting in a low nephron number [104]. In addition to exogenous administration, a developing fetus is likely to be exposed to excessive glucocorticoids of maternal origin (e.g., due to a stressed pregnancy). In rats, repeated dexamethasone administration on embryonic days 15 and 16 [97], from gestational days 16 to 22 [98], or from postnatal days 1 to 3 [99] was associated with reduced nephron numbers and resulted in hypertension in adult rats’ offspring.

### 3.7. Chronodisruption

Human studies have shown a link between gestational chronodisruption and adverse pregnancy outcomes [105,106]. In pregnant women, the disruption of circadian rhythms can occur through shift work, jet travel across time zones, or exposure to light at night [107]. A meta-analysis study recruiting 196,989 women reported that working rotating shifts is associated with preterm birth and small for gestational age (SGA), both risk factors for a low nephron number [106]. In rats, chronic photophase shifts throughout pregnancy program adult offspring to display renal dysfunction and hypertension [108].

## 4. Behind the First 1000 Days of Life

As summarized above, a diversity of environmental risk factors is associated with the programming of kidney disease during the first 1000 days of life. Based on what is now known about the magnitude of kidney development in the first 1000 days of life, it is not surprising that more work is needed to understand the underlying mechanisms behind the pathophysiology of kidney disease programming. A better understanding of these mechanisms will help in targeted therapy and prevention.

### 4.1. Mechanisms of Later Kidney Disease of Developmental Origin

**Figure 3:**
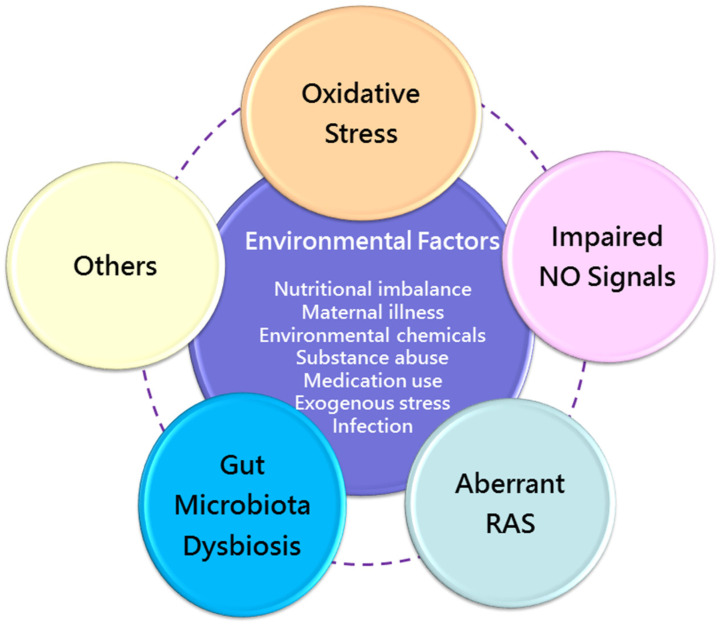
Overview of the common mechanisms of renal programming in response to various environmental risk factors during the first 1000 days of life. NO = nitric oxide; RAS = renin–angiotensin system.

Despite various early-life environmental factors related to CKD in later life, current evidence suggests that there may be common mechanisms behind renal programming. Although the complete mechanisms remain inconclusive, prior research has provided important information on certain molecular mechanisms, including oxidative stress [109], nitric oxide (NO) signaling [110], aberrant renin–angiotensin system (RAS) [111], and gut microbiota dysbiosis [112]. A summary of the integrated mechanisms of renal programming in response to various maternal insults for kidney disease of developmental origin is depicted in Figure 3.

Oxidative stress is considered to play a critical role in fetal programming [113]. Several mechanisms of oxidative stress have been related to renal programming, including the increased production of reactive oxygen species (ROS), antioxidant system dysfunction, and increased oxidative damage. As reviewed elsewhere, a number of animal models demonstrate oxidative stress involved in renal programming [105]. Nutritional imbalance during pregnancy and lactation is the most common factor to induce the programming of kidney disease. For example, calorie restriction [42] and increased consumption of a high-fat diet [40], fructose [114], or methyl donors [115] have been addressed previously. Other environmental factor associated with renal programming, such as environmental chemicals [73], substance abuse [82], maternal illness [50], inflammation [91], and medication use [97,98], have all been linked to oxidative stress.

A reduced nephron number induced by oxidative stress has been reported in the caloric restriction model [42], streptozotocin-induced diabetes [51], and maternal smoking [116]. As we reviewed elsewhere [94], renal programming induced by a variety of maternal insults is associated with increased F2-isoprostanes [49], malondialdehyde (markers of lipid peroxidation) [117], and 8-hydroxydeoxyguanosine (8-OHdG, an oxidative DNA damage marker) [49]. Conversely, the perinatal use of antioxidants has shown benefits against oxidative stress-induced renal programming in various animal models [109].

Renal programming, in addition to from oxidative stress, has been associated with impaired NO signals [110]. NO, a potent vasodilator, plays a key role in fetal development during pregnancy [118]. Nitric oxide synthase (NOS) catalyzes L-arginine to generate NO. However, in certain conditions, such as inhibition by NOS inhibitor asymmetric dimethylarginine (ADMA) [119], uncoupled NOS produces superoxide, consequently resulting in peroxynitrite formation. Accordingly, reduced NO bioavailability as a result of NOS uncoupling has been linked to kidney disease of developmental origin [110]. Moreover, our prior research showed that nephrogenesis was inhibited by ADMA, a ROS inducer, as well as an endogenous NOS inhibitor, consequently leading to a reduction in the nephron number [51].

Numerous interventions targeting the NO pathway in pregnancy to protect offspring against kidney disease have been reported [110]. These interventions include supplementation with substrates for NOS, NO donors, ADMA-lowering agents, and the enhancement expression/activity of NOS enzymes.

Similar to oxidative stress and NO, aberrant RAS appears to be involved in the pathogenesis of renal programming [115]. In the developing kidney, RAS genes are highly expressed and essential for mediating the proper formation of the renal structure and function [120]. Mutations in RAS genes are associated with kidney malformation in humans [121], which is in agreement with studies positing that the RAS is directly blocked by ACEI/ARBs [101]. Likewise, genetic inactivation of the angiotensinogen, renin, angiotensin converting enzyme (ACE), angiotensin II type 1 (AT1R) or type 2 receptor in mice leads to a broad phenotypic spectrum of CAKUT [121]. 

Angiotensin II (Ang II), the major player in the RAS, can mediate several key events of the inflammatory processes via AT1R stimulation [122,123]. These processes include triggering endothelial dysfunction, stimulating the release of cytokine/chemokines, activating NAD(P)H oxidase to produce ROS, and promoting pro-fibrotic growth factors, all contributing to kidney damage.

As reviewed elsewhere [111], most environmental influences that can program the kidney, resulting in adulthood kidney disease, such as nutritional imbalance, maternal illness, substance abuse, environmental chemical exposure, and medication use, are associated with the aberrant activation of the RAS. On the other hand, early blockade of the classical RAS axis appears to reprogram the inappropriately activated RAS to prevent the programming of kidney disease in various animal models.

Gut microbiota and their derived metabolites can affect the function of various target organs through circulation, including the renal systems [124]. Several adverse environmental factors in early life can shape the offspring’s gut microbial composition, leading to consequent adverse offspring outcomes [125]. Conversely, maternal microbiota-targeted interventions have shown benefits against renal programming [112,126]. Importantly, several gut microbiota-derived uremic toxins are associated with cardiovascular disease (CVD) in CKD via the activation of the aryl hydrocarbon receptor (AHR) [127]. Considering that several environmental factors (e.g., nutrition, environmental chemicals, and inflammation) are related to AHR activation, the interplay among gut microbiota, AHR, and the kidney has attracted the attention of researchers for investigating the mechanisms underlying the more thorough programming of kidney disease. Gut microbiota dysbiosis has been linked to hypertension by modulating the gut RAS [128]. On the other hand, ACE2, one component of the RAS, can mediate antimicrobial peptide secretion in the gut, leading to altered gut microbiota composition [129]. These findings indicate that there might be an interconnection between gut microbiota and the RAS behind the pathogenesis of renal programming.

Notably, environmental factors might display other potential mechanisms corresponding to renal programming, such as epigenetic regulation [130], dysregulated nutrient-sensing signals [131], and sex differences [132]. Although there are multiple mechanistic pathways outlined above, they might be interconnected to one another to drive renal programming, resulting in kidney disease. Better understanding the interaction between these common mechanisms and identifying new potential pathways to develop prevention interventions are key in the early prevention of kidney disease.

### 4.2. Prevention Actions

**Figure 4:**
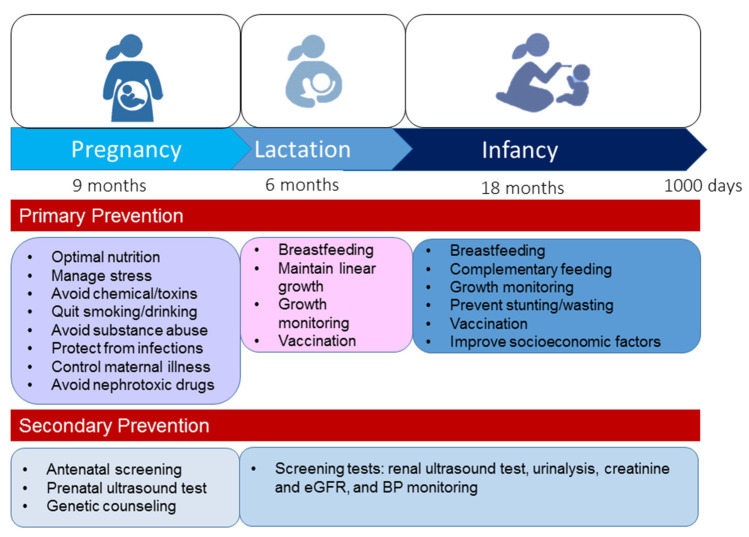
A summary of prevention strategies to improve kidney health during the first 1000 days of life.

In 2020, World Kidney Day informed the public about the importance of preventive interventions—primary, secondary, or tertiary [13]. In view of the complex nature of CKD, a holistic approach is required to positively impact kidney health. Tertiary prevention aims to manage advanced CKD and related comorbidities, which are rare during the early stage of life. Considering the prevention strategy from a DOHaD perspective, primary and secondary preventions seem to be the best strategy to improve global kidney health during the first 1000 days of life. Figure 4 illustrates the recommended primary and secondary prevention strategies from pregnancy to age 2.

First, primary prevention aims to prevent kidney disease before it occurs. The modifiable risk factors illustrated in Figure 1 should be avoided during the first 1000 days of life. During pregnancy through to early childhood, optimal nutrition is essential for supporting kidney health [133]. Neonates and young infants are particularly vulnerable to infections as they have naïve immune systems. As vaccination is one of the most cost-effective ways of preventing infection, enhanced early life immunity via taking necessary vaccines is essential to protect from infection [134]. Other key points are summarized in Figure 4. Moreover, additional attention is required to improve socioeconomic factors, e.g., access to family planning, equity and education for women, and reduction in poverty [7].

Secondary prevention suggests preventive measures that lead to screening for the early identification of disease and prompt treatment of kidney disease in the earliest stages. Although the early detection of CKD has the potential to yield marked public health benefits, most countries have inadequate CKD detection and surveillance systems to achieve this goal [135]. Some important services are required to screen for and detect kidney disease during the first 1000 days of life, such as antenatal screening, prenatal ultrasound testing, genetic counseling, renal ultrasound testing, urinalysis, creatinine and eGFR, and BP monitoring.

Considering CAKUT have a genetic basis, key nephrogenesis genes may form the basis of genetic screening tests for the future development of novel genetic therapies. In recent years, several potential biomarkers for the early detection of kidney damage have been introduced, and each of these biomarkers has advantages and disadvantages [136,137]. Nevertheless, currently, there is still no ideal biomarker of acute kidney injury. Furthermore, the search for an ideal biomarker predicting the progression of CKD in children with CAKUT is still ongoing. Although neutrophil gelatinase-associated lipocalin, and trefoil family factors (TFF) 1 and 3 have shown the potential to predict CKD progression in children with CAKUT [138,139], they still await more thorough validation. As precursor cell technology has been applied to generate new kidney tissues, more attention will need to be paid to the use of genetically altered metanephric precursor cells to differentiate into functioning kidney tissue for regenerative medicine therapies [140].

Given the advances in the DOHaD research field, it has become clear that kidney disease of developmental origin can be prevented in the earliest stage by reprogramming [11,12]. Prior animal studies have provided essential information in regard to reprogramming strategies. Considering that oxidative stress is a crucial mechanism implicated in renal programming, many natural antioxidants have been used as reprogramming strategies to prevent kidney disease in various animal models [109]: vitamin E and selenium [141], folate [142], L-taurine [143], L-tryptophan [144], N-acetylcysteine [48,49], resveratrol [73,74], and melatonin [44,100]. These findings support the notion that maternal nutrition is a double-edged sword for fetal programming: maternal malnutrition programs many NCDs, whereas nutritional intervention can also be advantageous to prevent adulthood NCDs [145].

Additionally, there are several reprogramming interventions targeting specific signaling pathways giving rise to benefits against renal programming. Targeting of the NO pathway in early life has been employed in various animal models to prevent the development of kidney disease in adult progeny. As reviewed elsewhere [95], these interventions include the supplementation of NO substrate, agents that lower ADMA, NO donors, and the enhancement of NOS expression. Likewise, RAS-based interventions have also shown promising results in protecting against renal programming and related diseases, such as renin inhibitor, ACE inhibitor, ACE-2 (ACE2) activator, and ARBs [111]. Furthermore, reprogramming interventions targeting the hydrogen sulfide (H_2_S) pathway [146] and nutrient-sensing signals [147] have also shown benefits with regard to kidney disease of developmental origin. Although significant advances have been made from animal research, the need for meaningful clinical translation remains a research priority.

## 5. Conclusions and Perspectives

Healthy people, living healthy lives on a healthy and peaceful planet were the ultimate goals stated by the United Nations in 2015, to be achieved by 2030 [148]. However, much remains to be accomplished to tackle the challenges of NCDs, in particular, kidney disease [5,6,7]. The concept of the first 1000 days of life allowed us to analyze the literature to determine the causes that could influence kidney development, identify the underling mechanisms of renal programming, and develop potential prevention strategies.

Though various modifiable early-life risk factors have been identified to date, preventive efforts should continue to discover other possible risk factors. Another important aspect is that current preventive strategies mainly focus on promoting a healthy lifestyle and avoiding exposure to risk agents. However, the translation of effective reprogramming interventions from animal studies into clinical practice has been far slower than expected. On all fronts, holistic and multilateral action is essential. Kidney health should be an imperative policy, which can be successfully achieved by the collaboration of doctors, nurses, allied health professionals, researchers, policy makers, and social workers. Only through collaboration can we implement not only patient but also global perspectives toward CKD prevention and the commencement of global kidney health futures in the first 1000 days of life.

## Data Availability

All data are contained within the article.
